# Pyoderma gangrénosum végétant idiopathique

**DOI:** 10.11604/pamj.2014.19.144.3012

**Published:** 2014-10-14

**Authors:** Karima Chakir, Badreddine Hassam

**Affiliations:** 1 Service de Dermatologie, CHU Ibn Sina, Rabat, Maroc

**Keywords:** Lésion végétante, pyoderma, gangrénosum, extrémités, idiopathique, vegetating lesion, pyoderma, gangrenosum, extremities, idiopathic

## Image en medicine

Le pyoderma gangrenosum est une dermatose inflammatoire rare faisant partie des dermatoses neutrophiliques évoluant vers des ulcères douloureux; trainants d’évolution torpide; on distingue la forme ulcéreuse; pustuleuse; bulleuse et la forme superficielle granulomateuse (végétante); il s'accompagne parfois de manifestations systémiques faisant sa gravité. Le pyoderma ganrénosum peut révéler une MICI; une maladie rhumatismale; une hémopathie maligne ou une néoplasie profonde. Par contre la forme végétante est le plus souvent idiopathique. Nous rapportons le cas d'un patient âgé de 45 ans de bas niveau socioéconomique; tabagique et éthylique chronique qui consulte pour des lésions ulcérées extensives des extrémités évoluant depuis 3 mois dans un contexte d'apyrexie; d'asthénie et d'altération de l’état général; le patient a été mis sous antibiotiques anti-staphylocoques sans aucune amélioration; l'examen dermatologique a objectivé une lésions bullo-hémorragique débutante ulcérée au niveau du dos de la main droite; des lésions ulcéro-végétantes exophytiques douloureuses multiples siégeant au niveau du dos de la main gauche; des poignets; des coudes; des chevilles; et de la région fessière confluentes par endroit en plaques arrondies et surmontées de croutes nécrotiques adhérentes; parfois à surface cribriforme; le reste de l'examen général était sans anomalie; les prélèvements bactériologiques et mycologiques négatifs; l'histologie a objectivé un infiltrat granulomateux et des abcès focaux à PNN ce qui a permis d’éliminer l'origine infectieuse et tumorale; ainsi on a retenu et le diagnostic de pyoderma gangrenosum végétant; le bilan n'a pas montré de localisation viscérale ou de maladie associée; et on a mis le patient sous corticothérapie et soins locaux avec bonne évolution clinique. Une surveillance clinique s'impose à la recherche d'une maladie associée qui ne peut apparaitre que plusieurs années après les lésions cutanées.

**Figure 1:**
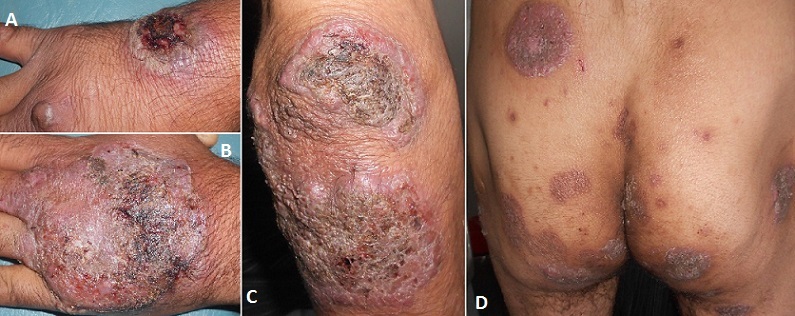
(A) lésion ulcérobulleuse débutante; (B) lésion végétante arrondie du dos de la main; (C) lésions végétantes fessières; (D) lésions ulcérovégétantes du coude

